# 

**Published:** 1885-07

**Authors:** 


Vol. VI.] TWO DOLLARS A YEAR. SINGLE COPIES 60 CENTS. [No. 3.

### THE JOURNAL

OF

### COMPARATIVE MEDICINE AND SURGERY.

### A QUARTERLY JOURNAL

OF THE

### ANATOMY, PATHOLOGY AND THERAPEUTICS OF THE LOWER ANIMALS.

*Edited by* { W. A. CONKLIN, Ph.D.

F. S. BILLINGS, D.V.S.

Entered New York Post Office as second class matter.

WILLIAM R. JENKINS,
Veterinary Publisher, Bookseller and Printer,
850 Sixth Ave., Cor. 48th Street,
NEW YORK.

LONDON: BAILLIERE, TINDALL & COX.

